# Assessing the influence of COVID-19 pandemic on thirty days readmission rate based on patient’s demographics, substance use, and insurance

**DOI:** 10.1080/07853890.2024.2399752

**Published:** 2024-11-04

**Authors:** Bashar Khiatah, Pooja Gajjar, Andrew Sous, Lara Aboud Syriani, Cindy Kim, Wisam Razook, Graal Diaz, Deborah Carlson

**Affiliations:** a Internal Medicine Department, Overlake Medical Center, Bellevue, WA, USA; b Internal Medicine Department, Loma Linda University, Loma Linda, CA, USA; c Internal Medicine Department, Community Memorial Hospital, Ventura, CA, USA; d Obstetrics and Gynecology Department, University of Loma Linda, Loma Linda, CA, USA; e Department of Medicine, Saint Elizabeth Medical Center, Brighton, MA, USA; f Department of internal medicine, University of Arizona, Tucson, AZ, USA; g Program Director of Internal Medicine Department, Community Memorial Hospital, Ventura, CA, USA; h Clinical Translational Researcher and Faculty, Graduate Medical Education, Community Memorial Hospital, Ventura, CA, USA

**Keywords:** Readmission, substance use, COVID-19 biasis

## Abstract

Providing the highest quality care with no biases is the goal of every healthcare system. As a part of that goal hospital readmission has been investigated due to its impact on healthcare cost and case fatality rate in patient outcome. Patient’s demographics, substance use and insurance barriers have been investigated as factors for readmission rate but the impact of COVID-19 pandemic on those biases and barriers have not been studied extensively yet. In our retrospective cross-sectional study, we placed the scope on readmission rate with the intention to investigate any relation with patient’s demographics including ethnicity, gender, language, substance use and insurance barriers and if there has been any change in the area pre and post COVID-19 pandemic. Total of 1713 readmitted patients were identified and split into 893 pre-COVID-19 and 820 post COVID-19 pandemic. Our multivariable analysis showed that the rate of readmissions during the COVID-19 pandemic was statistically higher among substance users (*p* = 0.003) and Medicaid insured (*p* = 0.038), and less likely among Spanish speakers *p* = 0.003. This study is limited due to small sample size and does not accurately represent the full population of the United States. Our hope is to further investigate the impact of COVID-19 pandemic on the readmission rate and to identify any influencing factors, biases, and barriers that contributed to the increased rate of readmission to learn and avoid future readmissions during straining times in medicine such as during COVID-19 pandemic.

## Introduction

Hospital readmissions are associated with poor patient outcomes, including increased morbidity and mortality and healthcare costs [1]. The rate of hospital readmissions can be an essential reflection of clinical quality. High rates portend concerns for low quality care and cause an increase in medical expenses [1]. However, multiple factors can play a role in having our patients go back to the hospital within a short period of time after their discharge. Benbassat et al. reported in their study that coordinating care between in-patient, outpatient, home, and community settings is crucial for high-quality health systems and decreasing readmission rates [2]. In an international study, the role of race and ethnicity in 30 days of readmission was assessed among diabetic patients, and it found that diabetic black patients had a statistically significant higher risk of readmission compared to members of other racial/ethnic groups [3]. Another study reported that patients with a non-English language preference have a higher readmission rate [4]. While many of these admissions could have been due to human biases, some are due to financial and administrative legislation set by the patient’s insurance system. This influence has been reported in a US-based study to evaluate the impact of insurance providers on patients’ destination upon discharge, which plays a substantial role in preventing readmission [5].

In California in 2019, 30 days readmission rate reached 14.9%, according to a California governmental study [6]. An estimated $25 billion is spent on preventable hospital readmissions that result from medical errors and complications, poor discharge procedures, and a lack of integrated followup care [6]. Thus, the U.S. government passed legislation (under the Affordable Care Act’s Hospital Readmission and Reduction Program [HRRP]) applying financial penalties for excess readmissions of Medicare patients [7]. Fortunately, quality improvement research can be used to reduce hospital readmission rates. Typically, these interventions involve system transformation [2]. Our research aims to study the difference in readmission rates pre and post the COVID pandemic at our institution in Ventura, CA. Moreover, our study investigates the role of a patient’s ethnicity, preferred language, health insurance provider, and substance use disorder in readmissions pre and post COVID-19 pandemic. This will help us understand if COVID-19 as a pandemic did impact readmissions overall or certain population’s demographic readmissions.

## Method

This is a retrospective cross-sectional study to determine if there were differences between pre and post-COVID readmissions (admission within 30 days of discharge from hospitalization) in a single academic healthcare institution. Our study used Electronic Medical Records (EMR) data from January 1, 2019, to December 31, 2021. All adult (>18 years of age) who were admitted to the hospital with a recent (within 30 days) discharge from the hositpal were included in the analysis. Other viraibales considered in this analysis were included that were reported by the patient such as gender, ethnicity, and religion. Other variables were: age, insurance primary carrier including commercial, medicaide or no insurance, substance abuse that was reproted from patient’s history from previous admissions for any form of drug abuse including but not limited to alcohol and opiates, patient’s status on a dmissions whether inpatient or observation, zip code of patient’s address and finally discharge status alive or dead. Descriptive and multivariable analyses (readmission visits) were performed using SAS 9.4 (SAS Institute, Inc.). The statistical significance level was set at (alpha) <0.05. This study was approved by our Institutional Review Board (IRB Number: 2021-HSR014).

## Results

Overall, there were *N* = 1713 readmissions (Table 1). Within these visits, *N* = 893 (47.87%) are pre-COVID, and *N* = 820 (52.1%) are post-COVID (*p* = 0.73). The studied population had equal representation of genders (50.03% female and 49.97% male, p-value = 0.91). Non-Hispanics accounted for 70.52% of the readmissions, and 29.48% were Hispanics (P-value = 0.001). The population consisted of 70.05% white; 29.4% other race; 1.93% black or African American; and, 1.52% Asian (P-value = 0.001). 10% spoke Spanish, and the rest (90%) spoke English (P-value = 0.001). Most readmissions were commercially insured (66.67%), 18.62% had Medicaid, and 14.71% had other insurance (P value = 0.01). The pre-Covid visits had higher proportions of Spanish-speakers and white race. Multivariable analysis showed that readmissions during the COVID-19 pandemic was statistically higher among substance users (OR = 1.86, CI = 1.23, 2.48, *p* = 0.003) and Medicaid insured (OR = 2.95, CI = 2.86, 3.03, *p* = 0.038), and less likely among Spanish speakers (OR = 0.53, CI = -0.11, 1.17, *p* = 0.003) (Table 2). Other covariates that were included in the model that did not meet significance level included gender, race, and ethnicity.

**Table 1.** Descriptive analysis of readmission visits pre (*n* = 893) and post-COVID pandemic (*N* = 820).

Variables	Pre -Covid Percent (%)	Post-COVID (%)	*P*-value
Readmission	52.10%	47.87%	0.73
Gender			
Male	50.05%	49.88%	0.91
Female	49.94%	50.12%	
Race			
White	78.27%	61.10%	0.03
Asian	1.57%	1.46%	
Black	1.80%	2.07%	
Other	18.02%	35.12%	
Insurance			
Commercial	36.84%	35.00%	0.64
Medicaid/Medicare	63.15%	65.00%	0.72
Substance Abuse			
No	94.00%	89.51%	0.85
Yes	6.00%	10.49%	0.08

The rate of hospital readmission decreased from 52.10% before COVID to 47.87% post-COVID, but the change is not statistically significant (NS). Both male and female populations experienced marginal shifts, with males slightly decreasing from 50.05% to 49.88% and females slightly increasing from 49.94% to 50.12%. However, these changes are not statistically significant (NS). Significant changes were observed in the racial composition. The percentage of White individuals decreased notably from 78.27% to 61.10%, with a p-value of 0.03, indicating statistical significance. Other racial categories like Asian, Black, and Other also showed some changes. The distribution of insurance types saw a slight decrease in the commercial category from 36.84% to 35.00%, but this change is not statistically significant (NS). Meanwhile, Medicaid/Medicare increased from 63.15% to 65.00%. Substance abuse increased from 6.00% to 10.49%, and the difference is not statistically significant (NS). However, there was a noticeable decrease in individuals without substance abuse issues from 94.00% to 89.51%.

**Table 2.** Multivariable analysis (model is for the number of readmission post COVID-19 pandemic).

Vairable	Level	Odds Ratio (OR)	Confidence Interval-low	Confidence Interval-high	*P*-Value
Substance Use	Yes	1.86	1.24	2.48	0.003
Patient Language	Spanish	0.53	−0.11	1.17	0.003
Insurance	Medicaid	2.95	2.86	3.03	0.038

*Note*. Significance level was set at <0.05. Only covariates that met significance level are reported in this table. Other covariates in the model that did not meet significance level included gender, race, and ethnicity.

## Discussion

This study reveals that readmission rates for patients with substance abuse, Spanish-speaking, and Medicaid-insured patients were higher during the COVID-19 pandemic; however, overall readmission rates were unaffected. While studies have evaluated the impact of bias on healthcare disparities, our study is a unique investigation of how implicit physician bias may interplay with patient demographics and the hierarchy of health insurance to influence readmission rates in the context of a global pandemic [8].

Hospital readmission results from many factors, from socio-demographic characteristics such as ethnicity, comorbidities, and patient living conditions to hospital length of stay [9]. Prior studies have demonstrated higher readmission rates for Black and Asian patients than White patients, suggesting the nuanced role of race [10]. The COVID-19 pandemic placed extraordinary demands on physicians leading to burnout and its sequelae of increased medical errors and poor quality of care [11]. Given the prevalence of implicit bias amongst physicians, our findings suggest that the pandemic may have augmented the influence of inherent bias as evidenced by the increased post-COVID readmission of the aforementioned patient populations.

Black and Latino-identifying patients face bias from providers across all specialties and stages of training [12]. We found that Spanish-speaking patients experienced higher readmission rates during the pandemic, which is consistent with previous research indicating that non-English speakers had higher readmission rates than English-speaking patients [4]. Despite the availability of digital interpretation services, ancillary healthcare staff often act as translators, which may compromise communication. Physicians may forgo the quality of communication instead of convenience due to potential implicit bias towards Hispanic patients and negative perception of health outcomes for this group. Regardless of the reason, communication barriers are detrimental to healthcare quality and patient safety [13].

Patients with substance abuse also experienced significantly higher hospital readmission rates in our study. Individuals struggling with substance use encounter considerable stigma from healthcare professionals, jeopardizing health outcomes [14]. This patient population has up to a 30% increased risk of leaving the hospital against medical advice (AMA), which invites a host of complications, including hospital readmission and increased mortality [15]. While leaving AMA and the consequent risk of readmission can be attributed to poor withdrawal and pain management, negative interactions with the healthcare staff are an essential contributor [16]. Patients with substance abuse feel stereotyped and marginalized by their providers, which pushes them away from completing the full duration of treatment [16]. Clinician bias is one potential explanation for our findings, given how pervasive stigma is against patients with substance abuse. Whether consciously or not, physicians may be less inclined to devote their full diligence to patients they believe to be responsible for their present affliction. The added challenges brought by COVID-19 may significantly amplify this bias.

## Limitations

We recognize that our study represents only a small sample of Ventura County of the state of California. Also, we recognize that an increase in substance abuse has been a nationally recognized during covid-19 pandemic that also could impacted our results. The substance abuse also was more prevelant among low socioeconomic status population who usually are medicaid insurance holder. On the other hand, the result related to the patient’s language is a strong finding as Ventura county has a large Spanish speaking population, that was impacted by COVID-19 pandemic and increased the readmissions rate. In order to appropriately assess the impact of social status, language barriers, insurance barriers on readmission rate and to recognize any biases based on these factors in a national wise conducted study is needed. This will assist providers across the United States to provide quality and care and to avoid readmissions which will improve the overall health and cut back substantially on the massive healthcare expenses in the United States.

## Conclusion

The COVID-19 pandemic did not impact the overall readmission rates in our community hospital. Sub-analysis of the data showed that during the COVID-19 pandemic, there was a significant increase in readmission rates for three populations: Spanish-speaking, Medicaid-insured, and substance abuse patients. While nationwide research is needed to investigate this increase in admission rates further, it is important to increase the inclusion of social work services, case management, and primary care followup in these populations to decrease the risk of readmission by providing more resources, help with the cultural, medical prospects, and have a tighter control of health-related risk factors increasing the risk of readmissions.

## Data Availability

Data is available upon reasonable request.
